# The Performance Analysis of the Map-Aided Fuzzy Decision Tree Based on the Pedestrian Dead Reckoning Algorithm in an Indoor Environment

**DOI:** 10.3390/s16010034

**Published:** 2015-12-28

**Authors:** Kai-Wei Chiang, Jhen-Kai Liao, Guang-Je Tsai, Hsiu-Wen Chang

**Affiliations:** Department of Geomatics, National Cheng-Kung University, 1 University Road, Tainan 701, Taiwan; kwchiang@mail.ncku.edu.tw (K.W.C.); cacalut1690@gmail.com (J.K.L.); tpp1114@gmail.com (G.J.T.)

**Keywords:** pedestrian dead reckoning, map aided, fuzzy logic, smartphone, indoor navigation

## Abstract

Hardware sensors embedded in a smartphone allow the device to become an excellent mobile navigator. A smartphone is ideal for this task because its great international popularity has led to increased phone power and since most of the necessary infrastructure is already in place. However, using a smartphone for indoor pedestrian navigation can be problematic due to the low accuracy of sensors, imprecise predictability of pedestrian motion, and inaccessibility of the Global Navigation Satellite System (GNSS) in some indoor environments. Pedestrian Dead Reckoning (PDR) is one of the most common technologies used for pedestrian navigation, but in its present form, various errors tend to accumulate. This study introduces a fuzzy decision tree (FDT) aided by map information to improve the accuracy and stability of PDR with less dependency on infrastructure. First, the map is quickly surveyed by the Indoor Mobile Mapping System (IMMS). Next, Bluetooth beacons are implemented to enable the initializing of any position. Finally, map-aided FDT can estimate navigation solutions in real time. The experiments were conducted in different fields using a variety of smartphones and users in order to verify stability. The contrast PDR system demonstrates low stability for each case without pre-calibration and post-processing, but the proposed low-complexity FDT algorithm shows good stability and accuracy under the same conditions.

## 1. Introduction

Over the past twenty years, several kinds of indoor navigation technologies have been developed based on various methods and theories [1]. This technological trend has changed and evolved quickly due to the growth of smart and wearable devices as well as advanced access points (AP). The ubiquitous smartphone has many kinds of internal micro-electro mechanical system (MEMS) sensors, such as Global Navigation Satellite System (GNSS chips), accelerometers, gyros, magnetometers, barometers and cameras. Those sensors are the basic components of some mainstream indoor navigation technologies such as pedestrian dead reckoning (PDR), image-aided PDR and radio frequency (RF)-aided PDR. Thus, the smartphone has the natural potential to become the ideal personal and mobile navigator. The number of people using their smartphones’ navigational services have increased significantly, spurring the development of location-based service (LBS) and precise personal marketing. However, the problems associated with using smartphones for indoor pedestrian navigation, such as the low accuracy of MEMS sensors, non-repeatable and unstable MEMS error characteristics, GNSS-denied environments, and the imprecise predictability of human motion, have limited the effectiveness and subsequent popularity.

PDR is one of the most common methods for using inertial sensors to estimate two-dimensional positions for pedestrian navigation. Given an initial position, PDR uses a pedometer, step length model and heading sensor to obtain positions rather than constantly producing integral calculations like the inertial navigation system (INS). Because the model simplifies the 3D world into a 2D world, the impact of gravity projection on horizontal components in INS is eliminated in PDR. However, PDR errors accumulate with each step in a similar manner to the INS errors that accumulate over time. The estimated heading of PDR usually depends on the integration of the gyro angle rate or calculation from a magnetic field. Both of these methods, however, also have their inherent problems. It has long been understood that gyros’ readings are strongly affected by human motion because of the vibration noise and dynamic misalignment between sensor body frame and pedestrian frame, causing error drifts quickly after integration. In regard to headings based on magnetic fields, magnetic materials and environmental obstacles degrade the magnetometer’s reading, negatively influencing the accuracy of the calculated azimuth. In addition to the accuracy of the heading, most of the step length estimations derived from empirical formulas fail to accurately meet users’ various individual characteristics. For example, step length can vary by as much as 40% with different pedestrians when they are walking at the same speed, and by up to 50% across the range of walking speeds of an individual [2]. Although tuning the parameters or calibration can enhance the accuracy in post-processing, it is inconvenient for real-time application. There is also the risk that the demonstration system performs better than the production system because of the uncertainty of the coefficients [3]. In conclusion, the PDR suffers error drift similar to that of the INS mechanization in terms of position or orientation because of the accumulated step-by-step impact of inaccurate step length and heading estimation. Therefore, in order for the PDR to maintain accuracy in individual cases, a PDR system requires pre-calibration or post-processing for tuning parameters similar to those in the Kalman Filter (KF) and step length model. However, this adjustment of parameters is time-consuming. Another method is using occasional position corrections from an external (absolute) positioning system to maintain stable performance [2]. Furthermore, PDR is a relative positioning system which needs an absolute coordinate for initialization.

Jeong-Min *et al*. propose an integrated system comprised of PDR and beacon-type AP to maintain stable performance [4]. The beacon-type APs’ system generates a Wi-Fi signal within a few meters of each AP as footprints where these APs are installed at the crossroad. With footprints covering all directions of a corridor, proximity is taken into account to obtain better accuracy than fingerprinting; this is accomplished by setting the threshold value of the received signal for triggering location-based events. The beacon-type Wi-Fi AP can be replaced by a Bluetooth beacon which can also determine positions using the proximity method. The newest version is Bluetooth 4.0, which includes the technology of Bluetooth Low-Energy (BLE). The broadcast of BLE avoids the channels that Wi-Fi generally uses, thus reducing interference. As the name suggests, it has lower power consumption (usually battery powered), low cost, lower air data rate, small size and easy distribution in any indoor environment, unlike the larger WiFi AP which needs to be placed near a power source. Faragher and Harle analyzed the accuracy of WiFi and BLE fingerprinting in a large indoor space with a highly-accurate ultrasonic ground truth referencing system [5]. They also analyzed the errors caused by the measurement noise, distance, walls and human bodies and concluded that the measurement of distance using BLE Received Signal Strength Indicator (RSSI) is very accurate within one meter because the signal strength decreases as the inverse square of the distance to the source. Thus, the proximity method eliminates the influence of multipath disturbance, the requirements of database construction and has strong performance within one meter; the battery-powered beacon is easy to distribute and the Bluetooth signal mitigates the signal interference. For these reasons, we settled upon a method using proximity and bounding box (for some initial positions that are far away from the proximity-type beacon) to the Bluetooth beacon to initialize at any position and update measurements instead of using Wi-Fi positioning and fingerprinting.

At present, maps and geospatial data are often used as an aiding source for navigation because of their reliability. They generally consist of nodes, polylines, polygons, individual IDs and attribute data in the format of XML or computer-aided drawing (CAD) files. The map formats become more consistent because of the development of the map-matching algorithm and standards of the map database; however, the traditional generation of a numerical map is time consuming and the matching algorithm seems complex. In addition, for such a crowdsourcing map database like OpenStreetMaps (OSM), there may be inaccuracies and precision problems, since the editors are not necessarily professionals. Thus, instead of using the aforementioned maps, the proposed system uses the reliable map surveyed by the self-developed Indoor Mobile Mapping System (IMMS). The IMMS quickly obtains the indoor map in the WGS84 coordinate system with high accuracy, depending on the advanced hardware and algorithm. The map format references OSM with some self-defined attributes. The theoretical basis of the map-matching algorithm generally includes topological relation [6], probability map, *etc*. The matching algorithm first projects the navigation solution onto the passageway or corridor both to constrain the error drift and provide boundaries to avoid crossing a wall. They use the previous position, velocity and heading from the navigation solution in combination with geospatial data to perform the required mathematics, such as determining minimum distance and weighted algorithms. Once these processes have been completed, the map matching decides the most likely location of the user. However, those traditional algorithms and mathematics are more complex and time consuming when they achieve higher accuracy. Link *et al.* propose a novel footpath-matching algorithm that integrates their indoor maps with OSM which is thus similar to a rule-based system. To be specific, the algorithm implements the matching based on the footpath which is a route that consists of steps and corresponds with a step-by-step heading [7].

**Figure 1:**
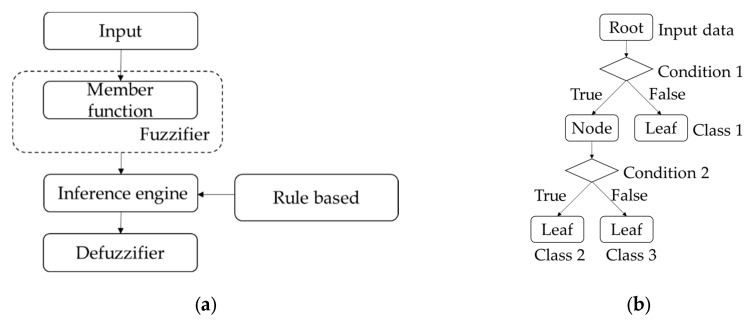
Two structures of decision making algorithms: (**a**) The fuzzy system includes fuzzifier, inference engine, expert rule and defuzzifer; (**b**) The binary decision tree includes the root, node and leaf.

Therefore, in order to combine map information, Bluetooth beacon and PDR with lower complexity, some logic algorithms have been applied in the attempt to further improve navigation accuracy. In the real world, many decision-making tasks are too complex to be understood quantitatively, but humans succeed by flexibly using knowledge that is imprecise rather than precise [8]. Realizing this, Zadeh first proposed the concept of fuzzy logic in 1965, thus launching a system that often provides more appropriate and simplified expressions for interacting with applied systems than can be achieved by their more exact mathematical model counterparts. Fuzzy logic uses imprecise, abstract and subjective knowledge to generate decisions or to map general numerical ranges for specific systems; it behaves like human reasoning and cognition, generating useful and usable answers without the need for deep understanding, exact equations, and precise numerical values. In other words, it is suitable for systems with mathematical models that are ill defined and difficult to derive, and it allows decision making with estimated values using simple, nonlinear equations and rules with *a priori* qualitative, uncertain and expert knowledge. Specifically, fuzzy logic expresses the input crisp system in a fuzzy set with a membership function in the fuzzifier; then, using an inference engine, it obtains the output of a corresponding fuzzy set using defined, knowledge-based rules. Finally, the defuzzifier transforms the corresponding fuzzy set into crisp values. The basic concept of the fuzzy system is illustrated in Figure 1a.

The application of fuzzy logic has dramatically increased in the last two decades, being used extensively in control theory, artificial intelligence, finance and other decision-making problems. Trawinski applied fuzzy logic based on FURIA-based and J48G-based multi-classification systems (MCSs) to improve the accuracy of a topology-based Wi-Fi signal strength fingerprint approach, where FURIA represents Fuzzy Unordered Rule Induction Algorithm and J48G is a simple decision tree [9]. Lai *et al*. estimated walking frequency and strength per step based on fuzzy logic for step length estimation in their PDR system [10]. Shahram *et al*. further propose a complete fuzzy logic architecture for step length estimation with the membership functions such as step interval, human locomotion, user’s height and trajectory curvature for the knowledge-based system (KBS) [11]. In looking at the literature, it becomes clear that fuzzy logic is used frequently for step length estimation and fingerprinting for pedestrian indoor navigation.

There are studies regarding fuzzy logic map matching for vehicle navigation. Haibin *et al.* propose an integrated map-matching algorithm based on fuzzy logic which combines a geometry map, probability map and the topology relation of the road network. Their fuzzy sets include three membership functions: projection distance, direction angle and comparability of trajectory [12]. Zhang and Gao have developed a fuzzy logic-based GPS system and map-matching integrated algorithm with fuzzy sets consisting of vehicle speed, heading error, perpendicular distance, gyro-rate and Horizontal Dilution of Precision (HDOP) for initial identification and tracking of the road link [13]. Working on similar problems, Wu has proposed a fuzzy sorting map-matching algorithm which generates road section candidates using a grid index, after which he uses a relativity function and fuzzy sorting method to determine the matching road section [14]. Ren and Karimi have even published a fuzzy logic map-matching algorithm for wheelchair navigation. They use fuzzy sets consisting of the perpendicular distance from a GPS point to a sidewalk and angular difference between the movement trajectories to build the necessary knowledge-based rules to produce the appropriate calculations [15]. Syed and Cannon have proposed a fuzzy logic map-matching algorithm with a high-sensitivity GPS receiver and a low-cost gyro for vehicle navigation in urban canyons. They designed two fuzzy inference systems which employ a variety of complete fuzzy sets to fix position and tracking [16]. Essentially, the strengths of fuzzy logic are its reduction of the depth and complexity of computations, improvement of accuracy and simplification of the positioning algorithm through the use of a relatively simple heuristic which determines a combination of the most appropriate rules, because an advanced indoor navigation system attempting to determine the necessary information through pure number crunching, with the required high computational complexity, would likely cause complete battery drain within a few hours. Keeping these problems in mind, this study focuses on developing a set of fuzzy rules based on the smartphone sensors and map knowledge for allowing individuals to successfully navigate an indoor environment in real time.

A decision tree is another low-complexity logic system for decision making; it consists of a hierarchical structure with a root, node and leaf, as shown in Figure 1b. Each internal node binary splits the dataset through the branches based upon the known conditions and features of the system; the classification will continue until a decision is produced at a leaf. The necessary conditions, or rules, can be obtained through a training process using sample datasets, and once generated, the new rules can be further used to make predictive estimations or system decisions. A decision tree based on Wi-Fi fingerprinting built during the off-line phase and used to determine a model for on-line application can be found in [17]. In addition, David *et al*. proposed methods based on the comparisons of RSSI and orientation information (magnetometer) to develop multiple weighted decision trees and data mining techniques for Wi-Fi fingerprinting [18]. There are also a variety of methods which use decision trees to aid in navigation, such as human behavior recognition [19] and Zero Velocity Update (ZUPT) detection. However, decision trees cannot solve problems of cognitive uncertainty or reduce classification ambiguity because of the need for exactly classified rules.

Therefore, some studies combine fuzzy logic and decision tree to improve the performance of a rule-based system. Umano *et al*. have proposed an understandable fuzzy decision tree (FDT) based on the Iterative Dichotmiser 3 (ID3) algorithm and defined fuzzy sets for the analysis of gas in oil [20]. In trying to overcome the obstacles of decision tree caused by binary splits for classification, Yufei and Michael introduced an FDT which is based on the reduction of classification ambiguity through the use of fuzzy evidence for classified application; they use linguistic terms with soft boundaries to accommodate vagueness and ambiguity in human thinking and perception [21]. Instead of having one class (leaf) from binary logic, the major difference between FDT and traditional decision trees (binary logic) is that the results may be members of more than one class after nodes/branches are calculated with fuzzy logic. Some mathematically oriented fuzzy decision trees are introduced in [22,23]. Inspired by the discovery of these various applications in the literature, we have taken the step of constructing a map-aided FDT for PDR to estimate the location of a pedestrian through the use of hierarchical rules and probability-based fuzzy sets.

Although complicated algorithms, various integrations, mathematics and multi-sensor fusion do indeed significantly improve navigation accuracy, these high-complexity approaches are more time/power consuming and require higher hardware standards. In addition, external complementary signals suffer problems, such as environmental changes and maintenance costs. Other simpler algorithms usually need the adjustment of parameters based upon post/pre-processing to adapt to each user, device and environment which is inconvenient for real-time application. Therefore, developing a stable, accurate and low-complexity system is a much more attractive direction for smartphone and real-time indoor pedestrian applications. Based on this idea, a system using map-aided FDT is proposed. The system determines the user’s location based on low-complexity rules, sensor data, fewer distributed Bluetooth beacons (depending on the floor plan’s geometry). The FDT rules are trained once using experimental field data. Then these rules are fixed in the map-aided FDT system for all other new experimental field tests. In other words, after the map survey by IMMS and one experimental file is used as trained data, the proposed system has the generalization ability to work on various individuals and smartphones. The difference between the proposed system and the contrast PDR system is the additional logic rules related to the map information.

There are three main advantages to this approach: (1) based on a single time training, the proposed system is versatile regarding different users, smartphones and experimental fields. In other words, the proposed system has the ability to work for new devices and individuals; in a crowd or a new environment such as a plaza (where it is hard to apply general map matching); and in corridors and a magnetic-hostile environment; (2) the system can achieve better stability without time-consuming pre/post-processing; (3) compared to traditional PDR, a low-complexity method, the proposed method is also a relatively simple while maintaining a similar level of error acceptibility without the need for continuous tuning. It is worth mentioning that for traditional PDR to achieve asimilar performance, anew tuning process is required for each users, sensor and environment.

## 2. Experimental Section

### 2.1. Indoor Map Production

**Figure 2:**
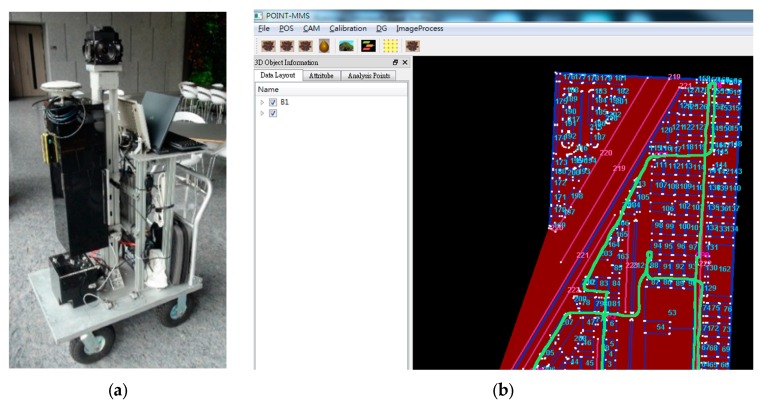
This figure shows the system and software for map production: (**a**) The hardware of IMMS; (**b**) The user interface of software for map production and measuring image.

IMMS was used to survey the building to generate a precise geo-referenced map, which is an important component in the proposed algorithm. The system is INS/GNSS integration and consists of GNSS, navigation-grade Inertial Measurement Unit (IMU), a panoramic camera, a power supply and an industrial computer. The use of this system reduces the time-consuming procedure for mapping a building and the result has high accuracy. A software was used to precisely estimate the trajectory of IMMS based on the Extend Kalman Filter (EKF) and Rauch-Tung-Striebel (RTS) smoother. The Non-Holonomic Constraint (NHC) and Zero Velocity Update (ZUPT) are used in the EKF. Information regarding these can be found in our previous research [24]. Another software was used subsequently to digitize and geo-reference the floor plan and then produce the walkable area in a .*csv* format file with WGS84 coordinates with some self-defined attributes. In addition, It also measures the point of interest (POI) based on the theories of photogrammetry and direct geo-referencing (DG) for the position of the installed Bluetooth beacon and its subsequent application. The coordinate of POI can be further applied to some applications such as route planning and investigation of the user’s hotspots. Those systems and software are part of an ongoing developed project at National Cheng Kung University (NCKU), as shown in Figure 2. Further details will not be discussed in this paper as a paper related to these systems will be published in the near future. Similar systems can be found in TIMMS [25].

### 2.2. Bluetooth Positioning

RF-based Bluetooth positioning is used for both initialization and update measurements during navigation. This experiment uses two methods, proximity and bounding box, implemented individually for each beacon with different setting IDs. Both of these methods are used to obtain the initial location, but only proximity is used for the update measurement. Some proximity-type beacons have heading constraint from map knowledge; the power level is one meter and is generally placed at the POI or corridor. When the system detects the signal from the beacons, the algorithm identifies their type based on their ID, and then decides on the next action. If the signal received is from the proximity-type beacon with an RSSI value larger than the threshold, then the algorithm obtains the coordinate and heading from the map database for initialization or updating. The signal strength distribution indoors is affected by the placement of the Bluetooth beacon and the environment [26]. To corroborate this idea, we have found that the proximity-type beacon may unevenly broadcast much longer compared to the setting power range (one meter in this study) because of the instability of RSSI, which may cause unexpected misdetection and a larger position error on the part of the proximity method, thereby rendering it unsuitable for placement in a plaza.

**Figure 3:**
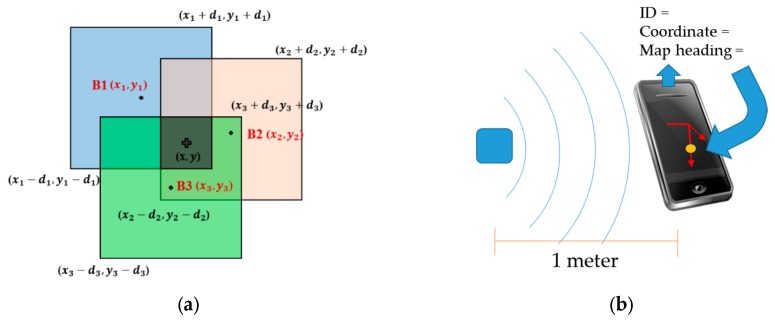
This figure shows the methods used in the proposed system for initialization: (**a**) The bounding box method; (**b**) The proximity method.

The bounding box method usually has a power level higher than ten meters, depending upon the field size, and beacons are placed in the corners of the plaza. Compared to the proximity method, initialization using the bounding box method is generally less accurate because of flaws in the RSSI-distance model, but it is more flexible in a large space. In addition, the heading used in the bounding box method relies on the magnetic heading because the initial location at the plaza has no corresponding heading in the map database. When the bounding box IDs are detected and the RSSI values exceed the defined threshold, the algorithm sorts their RSSI values and chooses the top three beacons, thus determining the coordinates according to their ID in map database for further position calculation. Each beacon produces a bounding box by the use of the beacon’s location ($x_{i},\;y_{i}$) as the square center with twice the distance (d) measured by RSSI-distance model as the edge length. In other words, the location of the vertex of the bottom left corner is ($x_{i}$ – $d_{i}$ ,$y_{i}\;$− $d_{i}$), and the location of the upper right vertex is ($x_{i}$ + $d_{i}$ ,$y_{i}$ + $d_{i}$). The user’s location, the intersection point of the squares, is defined by the following equations [27]:

(1) $$x_{max}=\max\left(x_{1}-d_{1},\;x_{2}-d_{2},\;x_{3}-d_{3}\right)$$

(2) $$y_{max}=\max\left(y_{1}-d_{1},\;y_{2}-d_{2},\;y_{3}-d_{3}\right)$$

(3) $$x_{min}=\min\left(x_{1}+d_{1},\;x_{2}+d_{2},\;x_{3}+d_{3}\right)$$

(4) $$y_{min}=\min\left(y_{1}+d_{1},\;y_{2}+d_{2},\;y_{3}+d_{3}\right)$$

(5) $$x=x_{\max}+\left(x_{min}-x_{max}\right)/2$$

(6) $$y=y_{\max}+\left(y_{min}-y_{max}\right)/2$$

where the equations are shown in a north−east coordinate system: ($x_{i},\;y_{i}$) is the i-th beacon’s coordinates as the center location of the square; $d_{i}$ is the measured distance between the user and beacon determined by the RSSI value; (x,y) is the point of the intersection. The concepts of proximity and bounding box are shown in Figure 3. By using the proximity and bounding box methods, the proposed system can initialize the user’s location almost anywhere, whether the user is in a large room or a narrow corridor; it also obtains some update constraints during navigation mode using a proximity-type beacon in conjunction with the map database.

### 2.3. Pedestrian Dead Reckoning

The general PDR was developed to compare with the map-aided FDT algorithm. It has two Kalman Filters (KF) for heading and position estimation. The heading measurements are derived from gyro ($A_{g}$) and the magnetometer’s readings ($A_{m}$). The characteristics of the gyro’s heading are smooth and independent of the environment. Nevertheless, errors accumulate quickly over time. In addition, the magnetometer provides the initial heading for the gyro’s heading as well as the heading derived from the magnetic field. The advantage of the magnetic heading is that it is independent of error drift. However, the magnetic heading is affected by the environment, which can cause unsmooth or even discontinuous measurements. The first KF estimates the heading as well as the gyro bias by fusing headings derived from the gyro and magnetometer. The fusion combines the advantages of both to obtain a smooth heading and reduce the error drift. The predictive stage of the first filter is shown in the following:

(7) $$x_{k}=\Phi_{k-1}x_{k-1}=\left[\begin{array}{c} \delta \psi_{k}\\ \delta b_{\psi ,\;k} \end{array}\right]=\left[\begin{array}{cc} 1 & \Delta t\\ 0 & 1 \end{array}\right]\left[\begin{array}{c} \delta \psi_{k-1}\\ \delta b_{\psi ,\;k-1} \end{array}\right]+w_{k},\;w_{k}\;\sim \;N\left(0,\;Q_{k}\right)$$

(8) $$P_{k}=\Phi_{k-1}P_{k-1}\Phi_{k-1}^{T}+Q_{k}$$

where *x* is the state vector; δψ is the heading error; δb is the gyro bias; $w_{k}$ is system noise assumed to be Gaussian distribution; Φ is the transition matrix which represents the relationship between the states at k and k−1 epochs; P is a covariance matrix of the state vector; Q is the covariance matrix of the system noise. The update stage is expressed as follows:

(9) $$K_{k}=P_{k}H^{T}{\left(\text{H}P_{k}H^{T}+R_{k}\right)}^{-1}$$

(10) $${\hat{x}}_{k}=x_{k}+K_{k}\left(z_{k}-Hx_{k}\right)$$

(11) $${\hat{P}}_{k}=\left(\text{I}-K_{k}\text{H}\right)P_{k}{\left(I-K_{k}H\right)}^{T}+K_{k}R_{k}K_{k}^{T}$$

where K is the Kalman gain; H is the design matrix for the measurement; $R_{k}$ is the covariance matrix of the measurements at k epoch; ${\hat{x}}_{k}$ is the updated state vector at k epoch; z is a measurement of the difference between the two kinds of headings in this case. The second filter uses the coordinates of the update measurement broadcast by the Bluetooth beacon (proximity type) to update the PDR position which is calculated based on the fused heading from the first KF and step length. The state vector and transition matrix are shown in the following equations:

(12) $$x_{k}=\left[\begin{array}{cc} \begin{array}{ccc} E_{k} & N_{k} & L_{k} \end{array} & \begin{array}{ccc} b_{E,\;k} & b_{N,k} & b_{L,k} \end{array} \end{array}\right]$$

(13) $$\Phi_{k-1}=\left[\begin{array}{cccccc} 1 & 0 & \vartheta & 1 & 0 & 0\\ 0 & 1 & \eta & 0 & 1 & 0\\ 0 & 0 & 1 & 0 & 0 & 1\\ 0 & 0 & 0 & 1 & 0 & 0\\ 0 & 0 & 0 & 0 & 1 & 0\\ 0 & 0 & 0 & 0 & 0 & 1 \end{array}\right]$$

where E is the local coordinate of the eastern direction; N is the local coordinate of north; L is the step length; ϑ and η are the second-order of Taylor series expansion for sine and cosine function; $b_{E,\;k}$ is the offset of positional error of east direction; $b_{N,\;k}$ is the offset of positional error in the northern direction; $b_{L,\;k}$ is the offset of step length. A detailed step length model is used from Ruizhi *et al*. and is shown as the following [28]:

(14) $$L_{k}=\left(0.7+a\left(H-1.75\right)+b\frac{\left(F_{k}-1.79\right)H}{1.75}\right)c$$

where $L_{k}$ is the step length of k step; a, b and c are the tuning parameters; H is the height of user; $F_{k}$ is the step frequency at k step. The pedometer is based on peak detection on the force of acceleration with the setting thresholds of peak value and time interval, as shown in Figure 4. Figure 5 shows the complete scheme of the contrast PDR system used in this study. The parameters of two KF and step length models were calibrated once based on data collected from one of the participants at the first experimental site (department building of NCKU) using a smartphone (HTC M7, HTC Corporation, New Taipei City, Taiwan). In addition, the rules of the proposed FDT system are trained once with the same data set.

**Figure 4:**
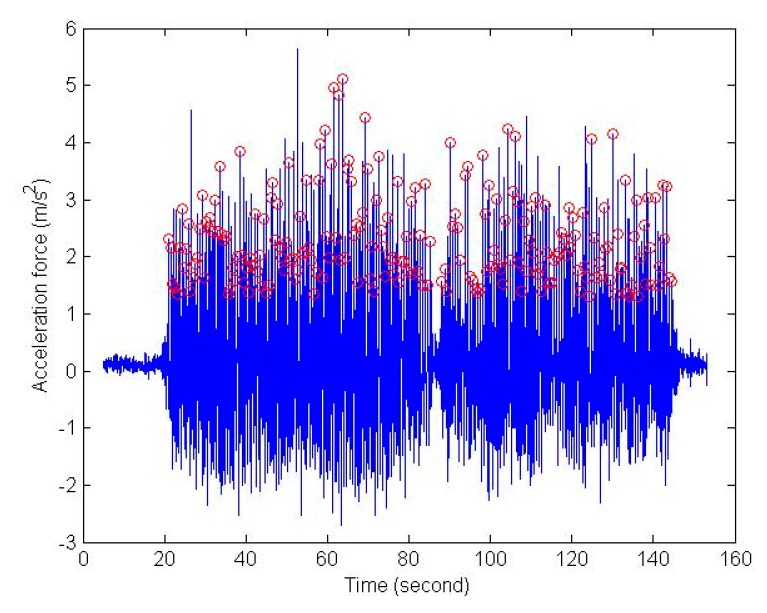
Example of peak value detection for pedometer.

**Figure 5:**
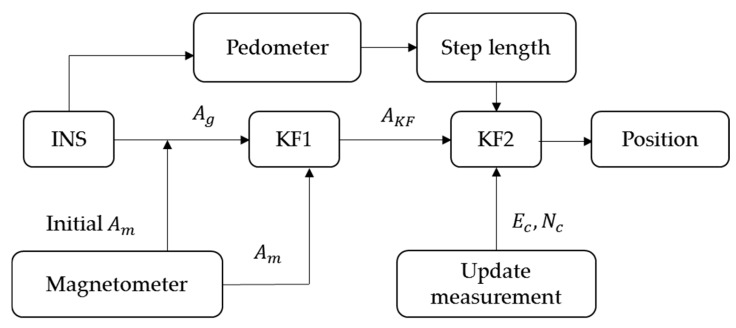
The contrast PDR system used in this study.

### 2.4. Fuzzy Decision Tree

**Figure 6:**
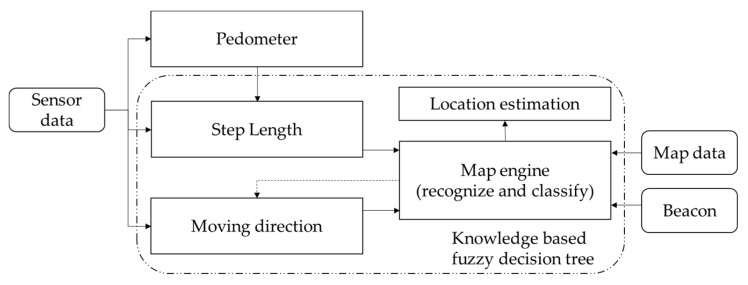
The architecture of the proposed system, with fuzzy decision tree.

The scheme for the proposed PDR system using a map-aided fuzzy decision tree is shown in Figure 6. The design of the pedometer is the same as in the contrast system described in Section 2.3. The map engine first loads the map data to construct a database with coordinates and their zone type. The zone type is one of the data attributes in the map database, which could be a corridor or plaza. The knowledge-based FDT has lots of fuzzy sets and rules inside. The fuzzy system can be easily shown in the following equation:

(15) $$A=\left\{\left(x,\;\mu_{A}\left(x\right)\right)\text{|}x\in X\right\},\;\mu_{A}:X\to \left[0,1\right]$$

where x is the object element; $\mu_{A}\left(x\right)$ is the fuzzy membership function; *A* is a fuzzy set; *X* is a crisp set. When the $\mu_{A}\left(x\right)$ is closer to one, it means the element *x* is more representative of the set *A*. The fuzzy decision tree shown in the Figure 6 has three fuzzy systems: step length, zone type and moving direction. The following paragraphs will respectively describe these systems.

**Figure 7:**
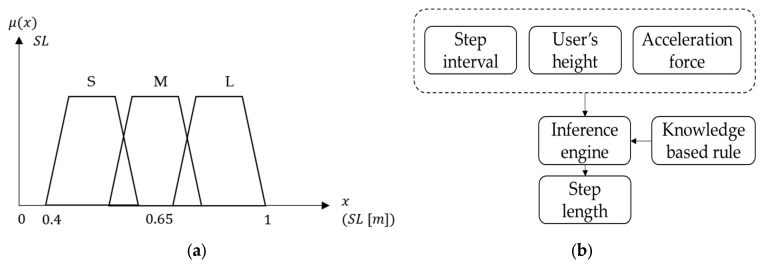
This figure shows fuzzy system of step length: (**a**) The membership function of step length; (**b**) The fuzzy system of step length with input of the three fuzzy sets.

The fuzzy system of step length can be seen in reference to the architecture proposed in [11], which is complete and detailed. Compared to this previous research, our design is simpler because we consider only one type of locomotion: walking. The membership function and fuzzy system of step length are shown in Figure 7. The membership functions of three fuzzy sets (step interval, user’s height and sensor measurement) are used in the inference engine, which uses knowledge-based rules to determine three levels of step length (short, medium and long). In addition, three fuzzy sets and each sub level have different weights in the inference engine based upon the knowledge system. The membership degree of each step length level is then determined in an inference engine, and the results are used as the input of a later decision tree. Table 1 shows the example of rules in the inference engine of step length, where the numbers represent the degree of each fuzzy level. As shown in this table, if the step interval is short, the user's height is tall and the acceleration force is large; step length is then short and the corresponding output degree is set to 0.6. Another case is when the step interval is short, user's height is medium and acceleration force is large, then step length is medium and the corresponding output degree is set to 0.4.

**Table 1:** The example of IF-THEN rules for the fuzzy system of step length.

IF	Activation Degrees	THEN	Output Degrees
Step interval is short AND user’s height is tall AND acceleration force is large	short 1, tall 0.8, large 1	Step length is short	0.6
Step interval is short AND user’s height is medium AND acceleration force is large	short 1, medium 0.2, large 1	Step length is medium	0.4

The map engine recognizes key information from the input map, such as the direction of a corridor and where the zone of the plaza is. In general, the corridors are perpendicular to each other in a building; therefore, the type of input map can be divided into vertical or oblique. The rules in the FDT algorithm must be slightly modified after the type of map has been decided, after which the map engine generates some map point candidates with zone type attributes around the previous location based on the levels of step length.

**Figure 8:**
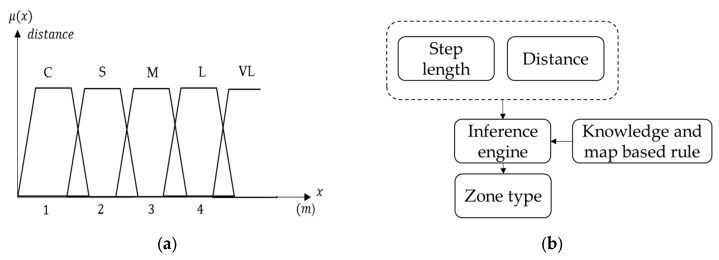
This figure shows the fuzzy system of the next zone type: (**a**) The membership function of minimum distance; (**b**) The fuzzy system of next zone type with the two inputs of fuzzy sets.

The membership probability and class of next zone type is determined by the inference engine, which uses the membership degree and level of step length and individual minimum distance. The minimum distance is determined by the previous position and the location of each zone. The weight of minimum distance is larger than step length, but step length has influence when more than one zone is close to the user. Thus, the classes of next zone type and their probabilities can be used in subsequent decision trees. The membership function of minimum distance and the fuzzy system of the next zone type are shown in Figure 8. Table 2 shows the example of rules in the inference engine of next zone type, where the minimum distance corresponds to each zone type; zone type 1 is a kind of corridor, zone type 2 is another kind of corridor perpendicular to type 1, and type 3 represents the plaza. The example in Table 2 continuously uses the assumption in Table 1.

**Figure 9:**
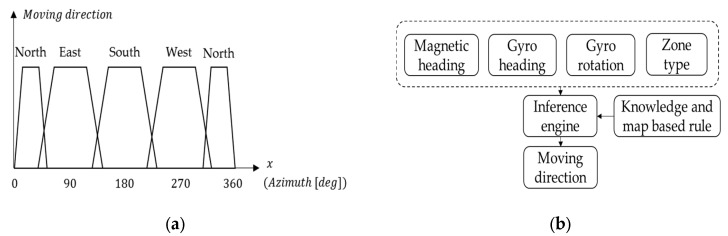
This figure shows the fuzzy system of the moving direction: (**a**) The membership function of moving direction; (**b**) The fuzzy system of moving direction with the input of four fuzzy sets.

The fuzzy system of moving direction is based on the magnetic heading, gyro measurement and map knowledge, which includes the next possible zone, such as the east−west corridor, as shown in Figure 9. Map-derived headings cannot be used exclusively since a corridor may be wide enough to have a wide range of possible directions. The inference engine gives their different weights through expert knowledge and map information. After the inference engine, the moving directions are divided into four or eight directions according to different current zone types (corridor or plaza). The inference engine also decides the probabilities of each direction as determined by knowledge-based rules, and then uses these results as the input data for a later decision tree. Table 3 shows the example of rules in the inference engine of a moving direction that, in keeping with previous tables, suggests that the magnetic heading may have interference, but the result is corrected based upon other fuzzy sets.

**Table 2:** The example of IF-THEN rules for the fuzzy system of next zone type.

IF	Activation Degrees	Previous Decision	THEN	Output Degrees
Minimum distance of zone type-1 is close AND minimum distance of zone type-2 are close and short AND minimum distance of zone type-3 is long	close 1 (type-1), close 0.4 (type-2), short 0.6 (type-2), long 1 (type-3)	Step length is short	Next zone type is type-1	1 (type-1)
		Step length is medium	Next zone type are type-1 and type-2	0.7 (type-1) and 0.3 (type-2)

**Table 3:** The example of IF-THEN rules for the fuzzy system of moving direction.

IF	Activation Degrees	Previous Decision	THEN	Output Degrees
Magnetic heading is east and north AND gyro heading is east AND gyro rotation is medium	east 0.2 (Mag.), north 0.8 (Mag.), east 1 (Gyro), medium 1	Next zone type is type-1	Moving direction is east and north	east 0.9 and north 0.1
		Next zone type is type-2	Moving direction is east and north	east 0.7 and north 0.3

**Figure 10:**
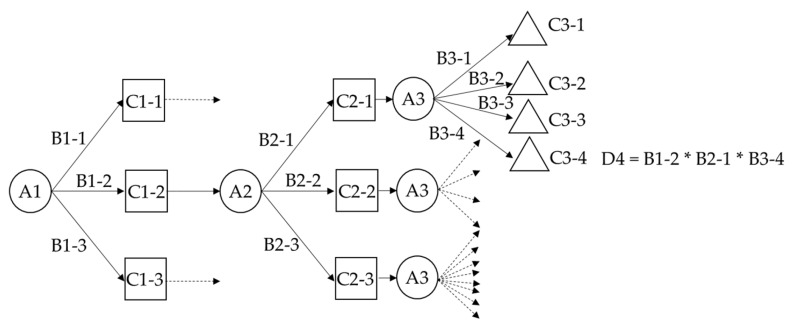
The fuzzy decision tree used in the proposed system with the knowledge-based rules and map information.

Instead of using a defuzzifier to obtain a crisp value for the set of the next zone type, step length and moving direction, the decision tree directly uses levels and degree (classes and probability) of the membership functions. If the three major components (next zone type, step length and moving direction) are collected, the decision tree calculates the final probability through individual branches. Each individual branch contains several nodes which decide the user’s location by multiplying the probability (degree) of each node on a specific branch. In other words, it chooses one of the locations from the four or eight candidates according to the fuzzy sets and their probability, as determined by the decision tree. The tree architecture shown in Figure 10 is simplified for illustrative purposes, where A1, B1 and C1 are the fuzzy set of step length, their membership degree and levels; A2, B2 and C2 are the fuzzy set of the next zone type, their membership probability and classes; A3, B3 and C3 are the fuzzy set of the moving direction, their membership probability and classes; D4 is the example of final probability from one of the branches. The plaza zone type has eight directions and another has only four, as it is a simple perpendicular corridor. Therefore, we can choose one of the map point candidates as the next user’s location based upon a direction which has maximum probability. Table 4 shows the example of the final decision tree which follows the previous tables after cutting off the branch of zero probability. The final decision is based upon the maximum probability of Branch 1.

**Table 4:** One of the possible situation in FDT system.

	Step Length (%)	Next Zone Type (%)	Moving Direction (%)	Final Probability (%)
Branch 1	Short (0.6)	zone type 1 (1)	east (0.9)	0.54
Branch 2	Short (0.6)	zone type 1 (1)	north (0.1)	0.06
Branch 3	Medium (0.4)	zone type 1 (0.7)	east (0.9)	0.252
Branch 4	Medium (0.4)	zone type 1 (0.7)	north (0.1)	0.028
Branch 5	Medium (0.4)	zone type 2 (0.3)	east (0.7)	0.084
Branch 6	Medium (0.4)	zone type 2 (0.3)	north (0.3)	0.036
	Final decision: Step length short, move to zone type 1 with *east* direction			
	Choose the corresponding candidate point			

The sample data was collected in a departmental building in the NCKU campus by one person using smartphone HTC M7. This experiment was designed to verify and modify the architecture of FDT and related knowledge-based rules. The main architecture following the test and various refinement procedures has three groups of rules, and some rules to avoid possible exceptions during the procedure are added. Then, the defined FDT system is fixed and applied to other test fields using different smartphones with map recognition and corresponding modification (such as the position of beacon and definition of direction). In conclusion, the scheme of the proposed FDT system was implemented in the following steps:

1. Recognize the map in order to choose the corresponding algorithm and database
2. Count the step through peak detection
3. Generate the map candidates around the previous location
4. Determine the class and probability of step length, next neighbor zone type and moving direction with fuzzy logic
5. Choose the most likely user’s location through the decision tree with the input of fuzzy membership value and knowledge-based rules.

### 2.5. Experimental Setting

**Figure 11:**
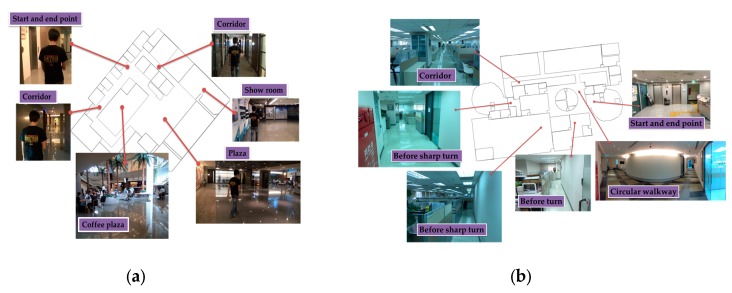
This figure shows the experimental environments of III and enterprise: (**a**) The environment of enterprise; (**b**) The environment of III.

The test smartphones are of different generations and include the HTC M7, SONY Z2 and SONY Z3 (Sony Corporation, Tokyo, Japan). Each smartphone has its own specification of inside sensors. Four participants, three males and one female, each held a smartphone in front of the torso and walked through the experimental routes. The characteristics such as height of each participant are different. The experimental fields include three different areas: the department building at NCKU, the office floor at the Institute for Information Industry (III) building and the hall of an enterprise. The area of the tested field at NCKU is about 1000 ${\text{m}}^{2}$, and includes classrooms and laboratories. The route begins at the end of a corridor and goes into a classroom and then back to the origin. The length of NCKU route is about 143 m. The area of the III tested field is about 2000 ${\text{m}}^{2}$, and contains offices with many computers and a circular walkway. The route starts at the elevator, which is a kind of POI, and goes around the office, and after some sharp turns and a circular walkway, goes back to the beginning. The length of the second experimental route is about 162 m. The third area is a hall at an enterprise field which is about 1600 ${\text{m}}^{2}$ at the ground floor. It is a typical company hall with many conference rooms and a coffee plaza, but there is no ceiling above the coffee plaza from the ground floor up to the second floor. In addition, there is an engine room on the third floor. The route starts at the elevator and proceeds along a corridor into a showroom and two plazas then goes back to the origin. The length of the third experimental route is about 175 m. Figure 11 shows the environments of III and enterprise. All the experimental fields are magnetic-hostile environments because of a lot of electronic equipment. Meanwhile, there are some spaces which are hard to determine the specific routes for with general map matching.

To precisely access the performance of the proposed FDT system, we have controlled the accuracy of the initial position by using proximity only. If the user starts the navigation in the plaza, the proposed system can still estimate the initial position using the bounding method. This method was not implemented in the test because its accuracy depends on the number of beacons, geometry of the distributed beacons and the degree of complication of the environment. For further information about initialization based on the bounding box method, refer to [29]. In order to show that the proposed FDT system can work in a harsh environment, frequent position updates and direction constraints from the proximity beacon were avoided. In the NCKU field test, only one proximity-type beacon was used to obtain the initial position. Other field tests have one beacon at the starting point for initialization and another one located near the beginning to correct the initial heading. The settings described in this paragraph are both used in the proposed FDT system and contrast PDR system.

## 3. Results and Discussion

**Figure 12:**
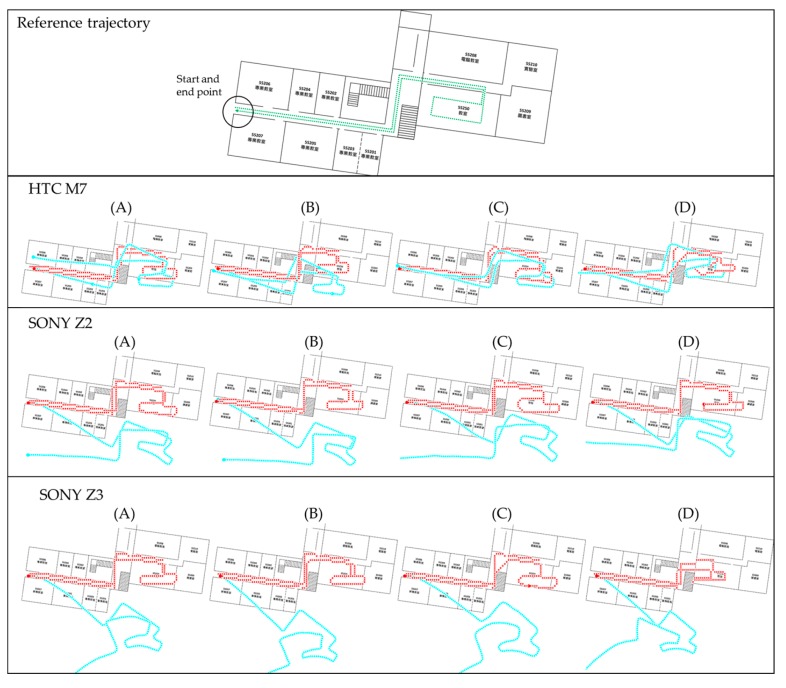
The experimental trajectories at *NCKU* department building.

**Figure 13:**
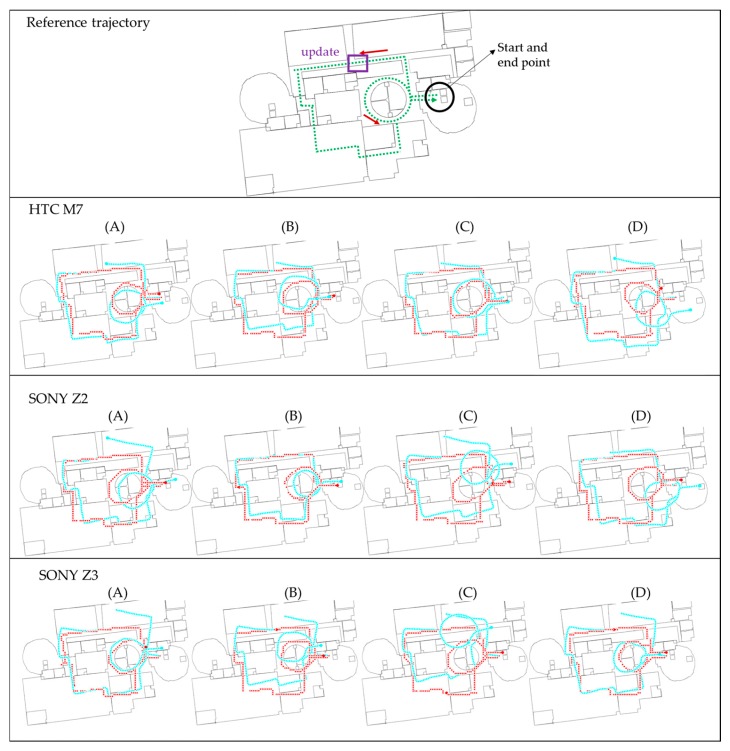
The experimental trajectories at *III* office floor.

**Figure 14:**
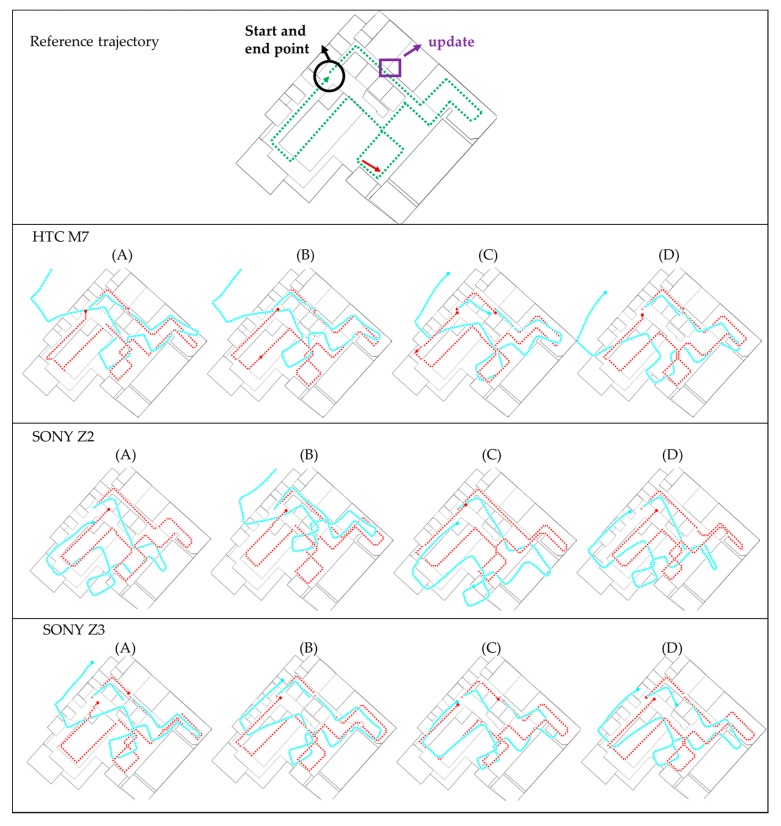
The experimental trajectories at the hall of enterprise.

The experimental results are presented in the following paragraphs. The developed Android software with proposed FDT algorithm was implemented to process the data in real time. Figure 12, Figure 13 and Figure 14 show the trajectories at NCKU, III and the enterprise, respectively. The green lines shown in the figures in the first row represent the real experimental route. This route is also a survey by the IMMS (using navigation grade IMU) which is estimated by the EKF and RTS-smoother with NHC and frequent ZUPT constraint. Because these two systems were not implemented together (time synchronization), no further position difference is shown. The red lines shown in the figures represent the proposed FDT algorithm, and the light-blue lines represent the contrast PDR system. The black circles indicate the starting and ending points. The purple rectangles indicate the locations where beacons were used to trigger the update operation. Symbols A, B, C and D represent the four different people in the experiments. In all figures, the second row shows the results of HTC M7, the third row shows SONY Z2 and the fourth row shows SONY Z3.

Figure 12 shows the experimental trajectories at NCKU department building. The result in the third of the second row is user *C* using the HTC M7, which is the sample data for tuning parameters and establishing knowledge rules used in the contrast PDR and proposed FDT systems, respectively. In other words, all the parameters or rules for both systems are only tuned once with HTC M7 and user C at NCKU field, and there is no further training or tuning for other smartphones, users and fields. Therefore, the other tests are designed to verify the stability and adaptability for real-time application.

The contrast PDR system as well as the FDT system performs well in the sample data because of the well-tuned parameters of step length model and KF in post-processing. Due to no update operation triggered during the navigation phrase, the contrast PDR system has no ability to correct the heading. Therefore, the contrast PDR performs worse in other cases at NCKU fields. Even if we correct the initial heading, the drift continues and clearly deteriorates the trajectory. It indicates that the parameters of contrast PDR in step length model and KF are not suitable to the different users and smartphones in a magnetic-hostile environment. Thus, the contrast PDR needs new calibration of the step length for different users and a new KF tuning process for different smartphones and magnetic fields in post-processing. Unfortunately, these procedures are time consuming and not suitable for real-time indoor navigation. In contrast, the proposed map-aided FDT system performs stably in real time at all the cases without further tuning requirement.

Figure 13 shows the experimental trajectories at III building which is an office with a lot of computers. In contrast to the previous NCKU field, there is a beacon (markedby a purple rectangle) to trigger the position and heading update near the beginning of the routes. Because of this beacon, the contrast PDR performs well at the beginning of all the cases, but still deteriorates over time. This phenomenon confirms that the parameters need individual calibration for each factor. However, the proposed FDT system still performs stably with good accuracy at III field. It is worth mentioning that the traditional topological map matching based on the polyline and nodes is hard to apply to this circular walkway, because the user may walk in a rectangular or a circular shape which cannot be matched with a fix route. The proposed system using the matching of eight directions can mitigate this problem.

Figure 14 shows the experimental trajectories at the enterprise building which has plazas, engine room and a high ceiling. There is also a beacon installed near the beginning to trigger the position and heading update. The contrast PDR still drifts over time because of a magnetic-hostile environment and inaccurate step length. The proposed system uses the matching of eight directions at the plazas without the specific route which is required by the traditional map matching. In addition, the RF-based positioning usually does not work in the high-ceilinged space or in spaces with serious electromagnetic interference caused by a large number of electronic devices. However, the results show the proposed FDT system still performs stably with good quality.

Table 5 summarizes the accuracy of all the results. The accuracy index is the percentage of loop closure error and total distance. For example, the accuracy of 1% means every one hundred travelled there will have one meter error accumulated. As demonstrated in Figure 12, Figure 13 and Figure 14, the proposed system has better performance than the contrast PDR system. Table 3 shows that the loop closure errors of the proposed FDT algorithm for all the experimental results and mean value is 1.3%. It is more accurate than the mean error of 8.21% in the contrast PDR. The standard deviation of the loop closure errors of the map-aided FDT system is less than 1%, which means it has high stability for different users, smartphones and fields.

**Figure 15:**
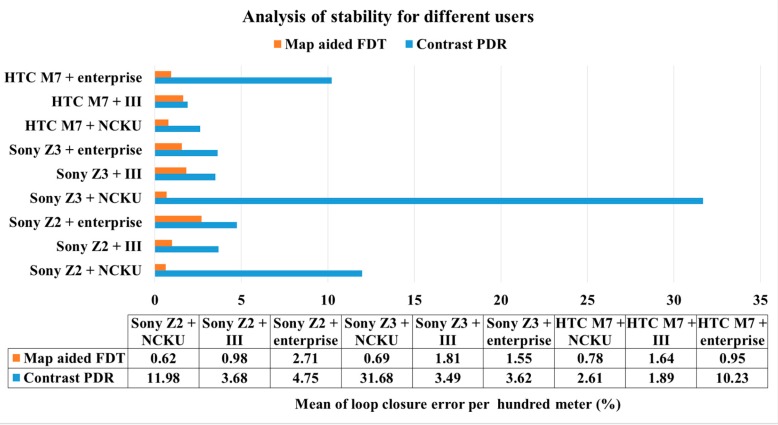
Analysis of stability for different users.

Figure 15 shows the mean values in terms of different users in the same field and using the same smartphone based upon Table 5. In other words, this figure indicates the stability of different users in the same situation. For example, the mean error of 0.62% at the first column represents the mean error of FDT system calculated from all users that use Sony Z2 at the NCKU field. The performances of contrast PDR are worse at the NCKU field because there is no update beacon at the beginning for initial heading correction. However, using the HTC M7 is better in this field because the parameters have been tuned. The figure shows that the map-aided FDT system has better stability when the user is different, regardless of smartphone or field.

**Table 5:** The accuracy of FDT and contrast PDR system.

Field	User	SONY Z2		SONY Z3		HTC M7	
		PDR	FDT	PDR	FDT	PDR	FDT
NCKU	A	12.07	0.61	29.96	0.66	2.43	1.09
143 m	B	14.00	0.66	33.44	0.68	1.30	0.74
	C	12.45	0.60	32.90	0.64	0.91	0.64
	D	9.40	0.61	30.42	0.77	5.79	0.66
III	A	3.19	0.89	1.84	3.86	1.28	0.93
162 m	B	1.98	0.99	3.28	1.33	0.78	0.61
	C	4.48	0.86	7.12	1.19	1.14	1.16
	D	5.07	1.17	1.71	0.87	4.36	3.87
Enterprise	A	3.47	3.39	6.99	1.66	13.03	0.54
175 m	B	9.91	2.31	3.47	0.89	11.12	1.19
	C	3.02	1.75	1.48	1.94	7.98	0.82
	D	2.59	3.38	2.53	1.71	8.79	1.25
	Mean of FDT: 1.3, Standard deviation: 0.94						
:	Mean of contrast PDR: 8.21, Standard deviation: 9.26						
	Unit: loop closure error/travelling distance (%)						

**Figure 16:**
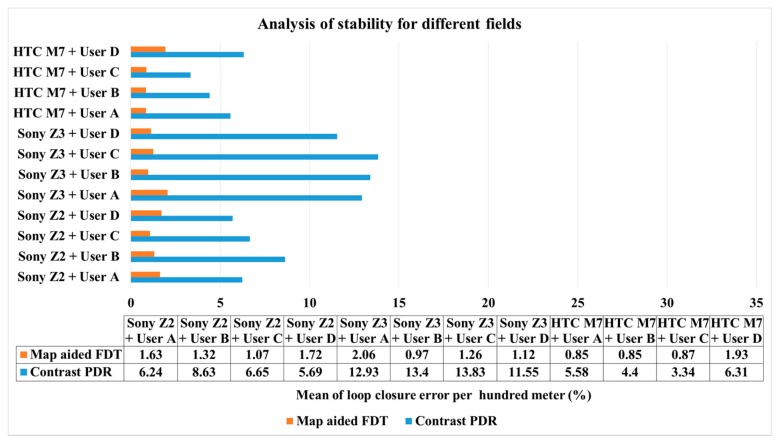
Analysis of stability for different fields.

Figure 16 shows the mean values in terms of the same user using the same smartphone at different sites based on Table 5, where it implies adaptability to the place. In other words, this figure indicates the stability of algorithms in different environments in the same situation. For example, the mean error of 1.63% at the first column represents the mean error of the FDT system calculated from the user *A* using Sony Z2 in all fields. The figure shows that the map-aided FDT system has better stability when the field is different, regardless of user and smartphone. In addition, because the parameters of HTC M7 have been tuned with user C at NCKU, the group of HTC M7 performs better than other smartphones. It is obvious that the parameters of HTC M7 are not suitable for other smartphones which have different specifications and need individual tuning to reach optimum performance.

**Figure 17:**
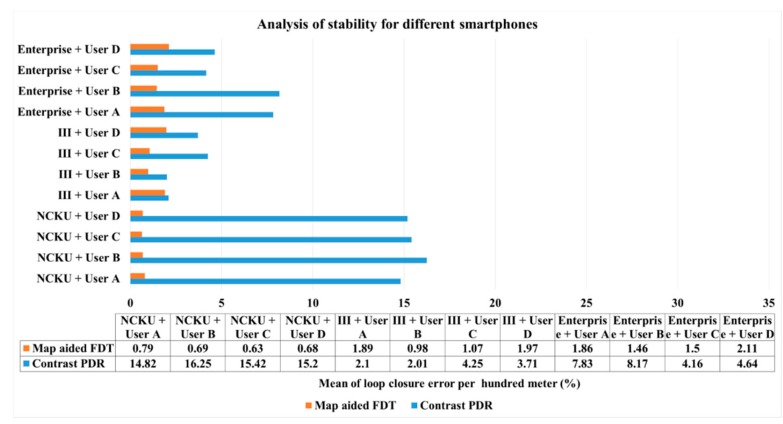
Analysis of stability for different smartphones.

Figure 17 shows the analysis for each smartphone when used by the same user in the same field, where it gives some indication of the acceptance of algorithms by the different devices. For example, the mean error of 0.79% in the first column represents the mean error of the FDT system calculated from user *A* using three kinds of smartphones at NCKU. The NCKU cases of contrast PDR are worse because there is no initial heading correction, but the NCKU cases of FDT are better than in other fields. The group of PDR values in III has better performance than the group of PDR values in enterprise, indicating that the different characteristics between two fields need an individual tuning process. This figure shows that the map-aided FDT system has better stability even when the specification of sensors is new to this system.

Ideally, the parameters and corresponding weights in KF of general PDR should be tuned for different fields and smartphones in order to compensate for the different levels of magnetic interference and differences within the sensor grade. This is because the accuracy of heading fusion depends on the accuracy of magnetometer and gyro as well as the parameters of the filter. The grade of accelerometers not only affects the pedometer but also indirectly influences the determination of step length. The model parameters of step length also depend on the characteristics of the user which needs calibration. According to these reasons, the contrast PDR system will perform poorly without individual pre-calibration and post-processing for each user, smartphone and field. However, the proposed FDT algorithm has better accuracy and stability in all cases without any further processing. It uses some simpler rules derived from one experiment and map information to mitigate the error from an inaccurate step length model and heading estimation. Therefore, the proposed map-aided FDT system provides stable average accuracy of about 1.3% based on 36 experiments in this study.

## 4. Conclusions

In order to simplify the necessary computations and time-consuming pre/post-processing needed in a practical indoor navigation algorithm, this research proposes an FDT algorithm which significantly improves the stability of pedestrian indoor navigation with an average loop closure error of about 1.3% as well as the standard deviation of about 0.94%. Even if some algorithms can achieve higher accuracy, they usually require a lot of time in pre-calibration and post-processing for individually tuning parameters such as the contrast PDR system in this study. The proposed system has better stability and adaptability without any further tuning for different users, places and smartphones. Furthermore, some systems achieve the same stability and accuracy based upon a complex algorithm, a large amount of calculation and various integrations for real-time indoor navigation. However, the proposed FDT system only uses the rules to integrate the map information and sensor data rather than topological or graphic matching and mathematical filter. The rule-based algorithm used has relatively low complexity because of the semantic logic conditions employed and many calculations reduced.

The map database and Bluetooth beacon are another important aiding source used in this study. Both the proposed FDT system and contrast PDR use the map database and Bluetooth beacon technique to enable both initialization and update measurements in real time. Although it is true that one can install more beacons to maintain navigational accuracy over a long time, the advantage of the FDT system is that it can provide acceptable accuracy even with only a small number of distributed beacons. Finally, the experiments performed for this research clearly demonstrate that the proposed FDT algorithm performs well for real-time pedestrian indoor navigation and also has high stability in different scenarios.
